# Diagnostic value of rapid on-site evaluation in interventional pulmonology: A protocol for systematic review and meta analysis

**DOI:** 10.1097/MD.0000000000021168

**Published:** 2020-07-17

**Authors:** Xiangwen Weng, Lijia Zhi, Xing An, Meixin Xu, Hua Zhang, Kunlan Long, Peiyang Gao

**Affiliations:** a Department of Critical Medicine, Hospital of Chengdu University of Traditional Chinese Medicine; b Clinical Medical College, Chengdu University of Traditional Chinese Medicine; c Respiratory department, Hospital of Chengdu University of Traditional Chinese Medicine, Chengdu, China.

**Keywords:** rapid on-site evaluation, interventional pulmonology, diagnostic value, protocol, systematic review, meta analysis

## Abstract

**Background:**

Rapid on-site evaluation (ROSE) is a kind of rapid evaluation of specimen satisfaction, preliminary diagnosis and priority strategy, the diagnostic accuracy of ROSE in the field of pulmonary intervention shows wide variation. The aim of the study was to further clarify the accuracy and diagnostic efficacy of ROSE in interventional pulmonology.

**Methods:**

This review summarizes and meta-analyzes studies of ROSE in interventional pulmonology, the ROSE diagnoses would be compared with the final pathologic diagnoses. The following electronic databases have been searched: PubMed, Cochrane Library, Embase, Web of science, CNKI, and WANFANG DATA. The methodologic quality of studies has been assessed using the Quality of Diagnostic Studies (QUADAS-2) instrument. This review is conducted using standard methods for systematic reviews of diagnostic accuracy studies. STATA SE 12.0 is used for data synthesis and analysis.

**Results:**

This review evaluates the accuracy and diagnostic efficacy of ROSE in interventional pulmonology, and the process factors that may influence the ROSE diagnosis are analyzed, such as Smear method, profession of smear technician, staining method, Profession of stain technician, Profession of reading slides, invasive procedure, Anesthesia method and etc.

**Conclusion:**

This review will stimulate proper evaluation of ROSE and provide assistance for clinical practice.

## 1 Introduction

Interventional pulmonology is an important branch of modern respiratory disease, which has been developing and making progress since the 1980s.^[1]^ Especially in recent years, with the development and application of high and new technologies, such as virtual bronchoscope, ultra-fine bronchoscope, endobronchial ultrasound (EBUS), electromagnetic navigation and etc., the success rate of interventional diagnosis of pulmonary peripheral nodules, mediastinum, pleura, and other difficult lesions by bronchoscopy was significantly improved.^[2,3]^

Rapid on-site evaluation (ROSE) is a kind of rapid evaluation of specimen satisfaction, preliminary diagnosis, and priority strategy when the specimen is collected by puncture, biopsy, brushing and so on, feedback on the techniques for guiding the next step. In theory, the use of ROSE can improve the diagnostic efficiency of interventional respiratory disease, reduce the operation time and the number of biopsy samples, and then reduce the occurrence of complications in interventional operation. However, ROSE has not been widely used in practice. Some observational Researches have shown that ROSE increases the yield.^[4,5]^ On the contrary, a meta-analysis of randomized controlled trials showed that additional use of ROSE neither improved the diagnostic yield nor reduced the procedure time during transbronchial needle aspiration (TBNA).^[6]^

In order to further clarify the accuracy and diagnostic efficacy of ROSE in interventional pulmonology, we conducted a systematic review and meta-analysis to compare ROSE with the final pathological results, extracted, and analyzed the factors that may affect the results of ROSE during the procedure.

## 2 Methods

This study has been registered in PROSPERO (http://www.crd.york.ac.uk/PROSPERO), registration number: No.CRD42020145807. The procedure of this protocol is based on PRISMA-DTA guidelines.^[7]^

### 2.1 Database and search strategy

The PubMed, Embase, Cochrane Library databases, Web of Science, Wan fang, and China National Knowledge Infrastructure (CNKI) were searched by combine keyword searches and thesaurus terms or subject headings as following: (lung OR pulmonary) AND (ROSE OR rapid on-site evaluation OR rapid onsite cytological evaluation) AND (diagnostic value OR sensibility OR specificity OR Negative predictive value OR positive predictive value).

The retrieval strategy for PubMed is as follows: ((((lung[Title/Abstract]) OR pulmonary[Title/Abstract])) AND (((((diagnostic value[Title/Abstract]) OR sensibility[Title/Abstract]) OR specificity[Title/Abstract]) OR negative predictive value[Title/Abstract]) OR positive predictive value[Title/Abstract])) AND (((ROSE[Title/Abstract]) OR rapid on-site evaluation[Title/Abstract]) OR rapid onsite cytological evaluation[Title/Abstract]).

### 2.2 Inclusion criteria

Studies meeting the following criteria were included:

1. The type of researches retrieved were not limited except case-control studies, case reports, abstracts, reviews, editorials, reviews, etc.
2. The subjects were required to sample the lesions through diagnostic interventional procedures without restrictions on auxiliary sampling equipment or technology in the process, such as radial endobronchial ultrasound (R-EBUS), fine-needle aspiration (FNA), transbronchial biopsy (TBB), Endobronchial ultrasound with a guide sheath (EBUS-GS), EBUS-TBNA, TBNA and etc. And ROSE should be used as an auxiliary intervention.
3. The results of each literature study can be listed in the form of 2 × 2 table, which shows the number of people who are divided into positive and negative according to ROSE diagnosis and final pathological diagnosis.

**Figure 1:**
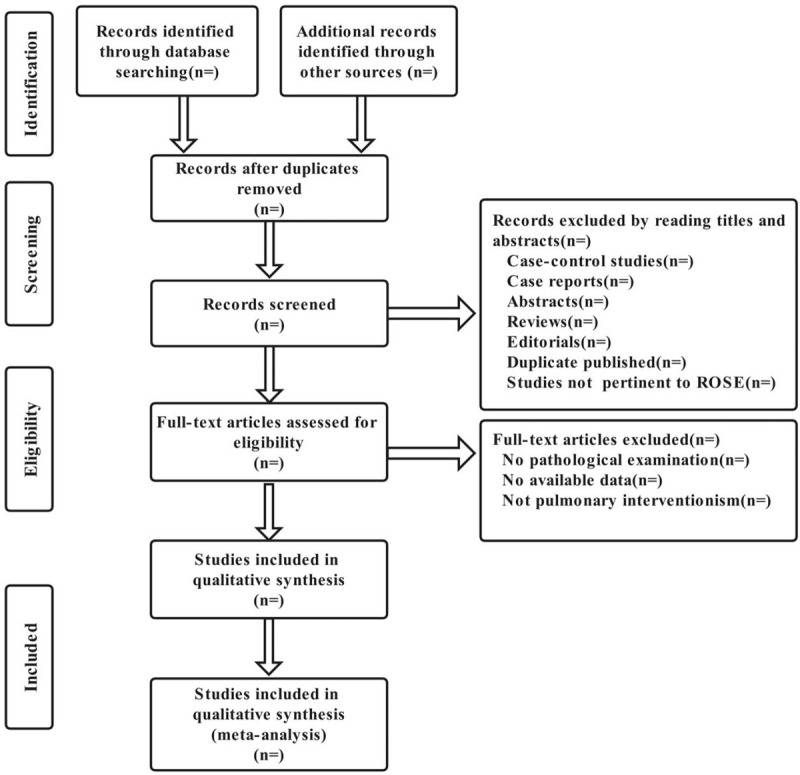
Flow chart of the selection process. ROSE = rapid on-site evaluation.

If the data cannot be extracted completely, the literature will not be included in the study. The selection process was showed in Figure 1.

### 2.3 Data extraction

Two researchers used Endnote literature management software to screen the included research according to the title and abstract content of the study. After screening, the literature was evaluated according to the inclusion criteria, we selected studies that met the requirements, and extracted and analyzed the data. The problems in the process of literature screening need to be discussed and solved by the 2 researchers. The following information was retrieved:

1. The basic characteristics of the literature: the first author, the country, the type of literature, the time of publication, the blind method, the gold standard, the number of research cases.
2. Main end point: the number of people who were divided into positive and negative according to ROSE diagnosis and final pathological diagnosis were extracted in the form of 2 × 2 table.
3. The factors that might have an impact on the main end point during the procedure: smear method, profession of smear technician, staining method, profession of stain technician, Profession of reading slides, invasive procedure, anesthesia method, sampling time (minutes), quantities of samples.

The 2 researchers eventually resolved any differences in the data extraction process through discussions.

### 2.4 Quality appraisal

Quality was evaluated using the Quality of Diagnostic Studies (QUADAS-2) instrument.^[8]^ The instrument consists of 4 components: Patient Selection, Index text (s), Reference standard, Flow and timing. Two researchers will independently evaluate each study for risk of bias and applicability. “L”, “H”, and “U” have been used as a code for the evaluations of the above bias risks.“L” indicates a low risk of bias, “H” indicates a high risk of bias, and “U” indicates the risk of bias is unclear. Disagreements resolved by discussion between all the researchers. When necessary, the study authors have been contacted to inquire some missing information. Trials of high risk of bias will be considered when conducting sensitive analysis.

### 2.5 Statistical analysis

For this diagnostic study, the estimates such as sensitivities, specificities were pooled by using a random-effects model. Heterogeneity between the studies was assessed using both the Q test and the *I*^2^ statistic. We considered an *I*^2^ value greater than 50% indicative of substantial heterogeneity. If we detect heterogeneity, Meta-regression analyses may be done to investigate the causes of heterogeneity, the sensitivity analyses would be conducted based on the study quality. In addition, Publication bias was analyzed through Deeks funnel plot test in midas. The whole statistical analyses were performed by Stata SE 12.0 (Stata Corporation, College Station, TX, USA), *P* values <.05 will be considered to be statistically significant.

## 3 Discussion

At present, ROSE technique has been widely used in the field of respiratory intervention, such as the pathological diagnosis of hilar and mediastinal lesions, peripheral pulmonary lesions, and Pleural lesions, but its clinical value and significance are not clear and controversial.

For instance, EBUS-TBNA can be used to sample hilar and mediastinal lesions that cannot be found under common bronchoscope, which has unique advantages in the diagnosis of hilar and mediastinal diseases and lymph node staging in patients with lung cancer. A large number of literatures have shown that ROSE technology combined with EBUS-TBNA or conventional transbronchial needle aspiration (C-TBNA) can reduce unnecessary puncture, reduce operation-related complications, guide the process of on-site operation, and improve the positive rate of diagnosis. Collins study found that combined TBNA and ROSE sampling reduced 33% of unnecessary punctures and 30% of unnecessary smears, due to the use of ROSE technology, 68% of the patients were successful with a single puncture of TBNA.^[9]^ In 2015, Cancer letters reported that 236 patients with lung cancer complicated with hilar and mediastinal lymph nodes were divided into 2 groups: ROSE group (122,252 lymph nodes) and non-ROSE group (114 cases, 260 lymph nodes). EBUS-TBNA was performed, and the diagnostic efficacy of the 2 groups was compared with the undiagnosed rate. The results showed that the undiagnosed rates of cytology and histology in the ROSE group were lower than those in the non-ROSE group, and the undiagnosed rates were 8.7% vs 14.6%, *P* = .038; 0.9% vs 4.4%, *P* = .018. This study suggests that the use of ROSE technology in EBUS-TBNA can reduce the rate of invalid samples and help to obtain positive diagnosis.^[10]^ Rokadia performed TBNA + C-ROSE examination on 625 patients with mediastinal enlarged lymph nodes with an average diameter of (14.4 ± 7.9) mm. It was found that the coincidence rate between ROSE diagnosis of hilar mediastinal benign granulomatous lesions and the final pathology was 81.6%. This study shows that not only malignant diseases, ROSE technology can also help hilar mediastinal benign lesions for on-site rapid diagnosis.^[11]^

As for peripheral pulmonary lesions, a prospective cohort study was conducted by Stein fort DP.^[12]^ Samples were collected under the guidance of R-EBUS. It was found that the time spent in C-ROSE group was lower than that in non-ROSE group (19 ± 8) minutes vs (31 ± 11) minutes, *P* < .0001). The positive rate of diagnosis in ROSE group was 97%, and there was no obvious complication of operation, so it was considered that ROSE + R-EBUS could help to improve the diagnosis rate of peripheral lung cancer, shorten the operation time, and reduce the risk of complications. In Chen study, R-EBUS guided transbronchial brushing or lung biopsies were performed in 815 patients with peripheral pulmonary lesions, of which 279 patients were randomly selected and received C-ROSE. The results of the study found that the diagnosis rate of peripheral pulmonary lesions in ROSE group was higher than non-C-ROSE group(88.9% vs 74.5% *P* < .05), suggesting that C-ROSE is helpful to improve the diagnostic efficiency of peripheral pulmonary diseases, the report also found that for some lesions in the tip of the right lung, the left tongue segment and other difficult parts, the diagnosis rate of ROSE group and non-ROSE group was 77.4% and 56.8%, there was still statistical difference.^[13]^ In pleural lesions, ROSE may also be beneficial. A prospective study showed that ROSE during medical thoracoscopy was found to have high accuracy for predicting malignancy. ROSE can provide the thoracoscopist with an on-site preliminary diagnosis, especially in cases with inconclusive macroscopic appearance.^[14]^ The above studies show that ROSE technique can help to obtain accurate and reasonable samples, improve the diagnostic efficiency of hilar and mediastinal lesions, peripheral pulmonary lesions and Pleural lesions, and reduce the risk of complications.

However, there are different views indicating that ROSE does not help to improve the positive diagnosis rate. For example, a retrospective study of Liu found that ROSE was helpful in guiding the operation of EBUS-TBNA, but did not improve the rate of pathological diagnosis of TBNA.^[15]^ Another study also found that ROSE helped to ensure the validity and adequacy of samples, and can provide optimal samples for flow cytometry, immunostaining, and molecular pathology, but it had no significant effect on the diagnosis rate.^[16]^ According to the study of Madan, in the 2 EBUS-TBNA groups, the use of ROSE had no significant effect on the diagnosis rate.^[17]^ In view of the large difference of the diagnostic value of ROSE, this review further clarify the accuracy and diagnostic efficacy of ROSE in interventional pulmonology. Besides, ROSE is a simple but complex program, the specific operation steps can be divided into 3 steps: production, dyeing, reading, and interpretation. Since the purpose of ROSE is to guide the intervention process in real time, its technical core lies in 2 points: Firstly, to improve the speed of film production and staining as much as possible; secondly, to accurately interpret the cytological results. So we extract and analyze the ROSE process information that might have an impact on the main outcome: such as smear method, profession of smear technician, staining method, profession of stain technician, invasive procedure, Anesthesia method and et al. We hope this review will stimulate proper evaluation of ROSE and provide assistance for clinical practice.

## 4 Others

### 4.1 Study status

This review is ongoing, the study has begun from August 2019 and it is expected to end in July 2020.

### 4.2 Founding

This study is sponsored by the science and technology department of Sichuan province (grant no: 2019YFS0084), the funder do not take part in the study design, data collection and analysis, or the preparation of the manuscript. The funder has provided only financial support for the study.

### 4.3 Ethics and dissemination

This review does not require ethical approval because the included studies are published data and do not involve the patients privacy. The results of this review will be reported in accordance with the PRISMA-DTA extension statement and disseminated to a peer-reviewed journal.

## Author contributions

**Conceptualization**: Xiangwen weng, Lijia Zhi, Xing An.

**Methodology**: Xiangwen weng, Lijia Zhi, Xing An.

**Software**: Meixin Xu, Hua Zhang.

**Supervision**: Kunlan Long, Peiyang Gao.

**Writing – original draft:** Xiangwen weng.

**Writing – review & editing:** Xiangwen weng, Lijia Zhi.
